# Factors associated with the severity of premenstrual symptoms in women with central obesity: a cross-sectional study

**DOI:** 10.1186/s41043-022-00343-5

**Published:** 2023-02-14

**Authors:** Payam Sharifan, Ali Jafarzadeh Esfehani, Amir Zamiri, Mansoureh Sadat Ekhteraee Toosi, Fatemeh Najar Sedgh Doust, Niloufar Taghizadeh, Maryam Mohammadi-Bajgiran, Hamideh Ghazizadeh, Fatemeh Khorram Rouz, Gordon Ferns, Majid Ghayour-Mobarhan

**Affiliations:** 1 Department of Nutrition, School of Medicine, Mashhad University of Medical Sciences, Mashhad, Iran; 2 International UNESCO Center for Health-Related Basic Sciences and Human Nutrition, Mashhad University of Medical Sciences, Mashhad, Iran; 3 Metabolic Syndrome Research Center, Mashhad University of Medical Sciences, Mashhad, Iran; 4 Department of Biology, Faculty of Sciences, Mashhad Branch, Islamic Azad University, Mashhad, Iran; 5 Student Research Committee, School of Medicine, Mashhad University of Medical Sciences, Mashhad, Iran; 6 Division of Medical Education, Brighton and Sussex Medical School, Brighton, UK

**Keywords:** Premenstrual syndrome, Risk factors, Nutritional status, Depression, Psychometrics

## Abstract

**Introduction:**

Premenstrual syndrome (PMS) is a common condition that affects social and psychological well-being of women. The risk of PMS is higher among obese women. The aim of this study was to identify the factors that influence the severity of PMS in women with central obesity.

**Materials and methods:**

This cross-sectional study was performed on 30–50 year-old women with abdominal obesity (waist circumference > 80 cm). The following data were collected: demographic data, anthropometric measurements, premenstrual symptoms screening tools, semi-quantitative food frequency questionnaire, 42-item depression, anxiety, and stress questionnaire (DASS-42), as well as serum vitamin D, and renal function tests.

**Results:**

A total of 139 women (mean age of 41.40 ± 7.39 years old) participated in the study. The prevalence of mild, moderate and severe premenstrual symptoms was 38.7% (55/142), 31.7% (45/142) and 27.5% (39/142), respectively. There was no significant difference between the groups in terms of anthropometric measurements and energy-adjusted nutrient intakes (*p* > 0.05). There was a significant relationship between moderate PMS and energy-adjusted saturated fatty acid (SFA) (*p* = .018, OR = .010 and 95% CI for OR: < .001 and .452), and energy-adjusted riboflavin (*p* = .042, OR = .005, 95% CI for OR: < .001 and .821), and between severe PMS and age (*p* = .034, OR = .906, 95% CI for OR: .826 and .993), and energy-adjusted monounsaturated fatty acid (MUFA) intake (*p* = .041, OR = 23.789, 95% CI for OR: 1.138 and 497.294).

**Conclusion:**

High intakes of MUFA and younger age were associated with a greater severity of PMS, while riboflavin intake was associated with reduced PMS severity.

## Introduction

Premenstrual syndrome (PMS) is a collection of affective and somatic disorders that young women commonly and constantly face in the luteal phase of their menstrual cycle, especially before menstruation [1, 2]. PMS severity differs between women and appears to be associated with their psychological, environmental, social, and physiological factors [3]. PMS is associated with impaired health- and work-related quality of life and increased need for healthcare intervention. In addition, this condition may interfere with their relationships and daily activities [4]. According to a recently published meta-analyses, the overall global prevalence of PMS is 47.8% [5]; however, the study did not include prevalence articles from the United States. Nevertheless, the prevalence of PMS is higher among Iranian women (71 percent) compared to the global prevalence [6]. It is hypothesized that nutrition, physical activity level, age, and the use of differences PMS measurement tools might be the reason for the regional differences in the prevalence of PMS [6, 7].

Many studies have evaluated the factors associated with PMS and reported that age, smoking and alcohol consumption; low consumption of fruits, sea foods, plant proteins, and high intake of whole and refined grains, high fat and overall calorie intake, as well as low socioeconomic status, being single, and familial history of PMS were related to severe PMS symptoms [8–10]. It was also reported that inflammatory biomarkers including interleukin-4 (IL-4), IL-10, IL-12, and gamma interferon (IFN-γ) could significantly rise in cases with PMS compared to controls [11].

Obesity is a global health problem with a growing prevalence [12]. The global prevalence of obesity is estimated to be 39% and is found to be more prevalent among women [13]. Obesity has long been considered as a risk factor for ischemic heart disease, cancer and stroke [14]. Obesity is a risk factor for PMS [15–18]. This association with PMS may be due to hyper-androgenism, as well as social, behavioral and neural effects of adiposity on female body [15, 19, 20]. The most frequent comorbidity of obesity in women is polycystic ovarian syndrome (PCOS), which is mainly due to insulin resistance and results in abnormal menses, hyper-androgenism and ovarian cysts [21]. The prevalence of PMS is high among women PCOS [22]. Obesity is also associated with inflammation, which might be associated with PMS [23, 24]. It was previously shown that women with mood disorders, or PMS consumed more carbohydrate-rich foods, which may result in adiposity [10, 25, 26]. Previous studies have been inconsistent in terms of the prevalence of PMS among overweight or obese women. While some studies indicated obesity especially visceral obesity were associated with increased PMS severity [27–29], other studies found that obesity was not associated with PMS [30, 31]. Therefore, obesity may be a risk factor or complication of PMS.

Although various studies on different populations have investigated the relationship between nutritional, biochemical, and psychological factors on PMS, there is scarcity of evidence regarding the relationship between the combination of these factors and PMS. Furthermore, as these parameters are affected by region and race, evaluating their relationship with PMS should be considered based on the geographical characteristics of the population. Therefore, we aimed to assess these nutritional factors in association with the severity of PMS on a sample of Iranian population in order to know more about the exact mechanism of PMS symptoms.

## Methods

### Study design and subjects

This report is a subanalysis of the *Survey of Ultraviolet intake by Nutritional Approach* (SUVINA study), conducted from January to March 2019. Details of the SUVINA study have been published elsewhere [32]. Data of 143 women were selected from the 346 adults who were screened for eligibility to participate in the SUVINA study. Inclusion criteria were as follows: women between 30 and 50 years of age who were not postmenopausal, had regular menstrual cycles (menstrual cycle duration between 21 and 35 days with menstruation for up to 7 days) at least in three consecutive cycles, and were willing to complete questionnaires to provide data for the study. Exclusion criteria were: pregnancy or lactation; any underlying diseases, including irregular menstrual cycles, or amenorrhea due to the reproductive dysfunctions.

Ethics approval was obtained from the National Institute for Medical Research Development (NIMAD; protocol ID: IR.NIMAD.REC.1396.027) and the Mashhad University of Medical Sciences (MUMS) research committee. Informed consent was obtained from all participants after being informed about the confidentiality of the collected data. In this regard, name or ID of the participants were not recorded but instead, participants were given unique codes. Only the main investigator was aware of the identity of the participants. Participation was voluntary, and participants were informed about the study methods.

### Data collection and questionnaires

All participant completed a socio-demographic questionnaire. Anthropometric indices, including weight, height, waist circumference (WC), hip circumference, neck circumference, and waist-to-hip ratio were evaluated by an experienced staff. Body mass index (BMI) was calculated for each participant by dividing body weight (kg) by the square of height (m). WC was measured by a nonelastic measuring tape while standing at midpoint between the lowest rib and the iliac crest [33]. Abdominal obesity was defined as WC greater than 80 cm [34]. Also, blood pressure for all participants was measured by one expert physician at sitting position after 20 min of rest.

A validated Persian version of premenstrual symptoms screening tools (PSST) questionnaire was used to assess the severity of premenstrual symptoms [35]. This questionnaire contains of 19 items in two main categories: (1) physical, psychological, and behavioral symptoms (the first 14 items) and (2) the impact of symptoms on the life functioning of women (the last 5 items). Items were scored based on a four-point Likert scale (not at all = 0, mild = 1, moderate = 2, severe = 3). The severity of premenstrual symptoms is categorized based on the sum of PSST scores into mild (0–19), moderate [19–28], and severe (> 28).

A semi-quantitative food frequency questionnaire (FFQ) containing 65 food items was used. The FFQ was validated previously (Cronbach’s alpha = 0.67) [36]. Consumption of each food item was evaluated in 5 frequency groups and portion sizes. An expert nutritionist was responsible to complete questionnaires. Consumed portion sizes were converted into grams. Nutrition composition assessment was performed using the Nutritionist 4 software. The United States Department of Agriculture food composition table values were applied for local Persian foods.

The 42-item depression, anxiety, and stress questionnaire (DASS-42) was used to evaluate the mental health of the participants in the mentioned three subscales. Each scale consists of 14 items. Depression subscale evaluates dysphoric mood types such as depreciation, low self-esteem, despair, lack of motivation. Anxiety subscale evaluates arousal state, like autonomic arousal, muscular rigidity, and anxious affect. Stress subscale evaluated nervous tension and irritability. Persian validation of this questionnaire for the Iranian population was previously reported [37].

### Laboratory measurements

In the main study, blood samples were taken from participants after a 12 h fasting in the morning. Venous blood samples were collected into plain Vacutainer^®^ tubes. Samples were then centrifuged at 5000 g for 15 min at 4 °C to separate the serum and aliquots of serum stored frozen at − 80 °C for future analysis. In the current study, total serum vitamin D values were recorded and analyzed. Total serum 25(OH)D concentrations were assessed using a commercial enzyme-linked immunosorbent assay (ELISA) kit (Pishgaman Sanjesh-Iran), using an Awareness/Stat Fax 2100 analyzer.

### Statistical analysis

The statistical package for social sciences (SPSS) software version 16 (IBM Statistics, Chicago, Il, USA) was used for data analysis. The dietary intake of nutrients was adjusted for energy intake based on residual method [38]. In order to obtain energy-adjusted nutrient intakes, absolute intake of each nutrient was entered into a linear regression model as dependent variables and energy intake as an independent variable. The residuals were used to calculate the adjusted nutrient intake by adding the residual values to the mean intake of the nutrient. One-way analysis of variance (ANOVA) was used to compare continuous variables between premenstrual syndrome severity groups. The Tukey test was used as the post hoc test. The chi square test or Monte Carlo tests were used to compare the distribution pattern of categorical variables between premenstrual symptom severity groups. Linear regression was performed to assess the relationship between PSST score and study parameters using PSST score as dependent variable and study parameters as independent variables. The backward model was used for the analysis. The level of statistical significance was *p* < 0.05 in this study.

## Results

The mean age of the participants was 41.40 ± 7.39 years old. Demographic characteristics of study subjects are presented in Table 1. The mean and SD for PSST scores of the participants were 20.46 ± 12.05. The prevalence of mild moderate and severe premenstrual symptom were 38.7% (55/142), 31.7% (45/142) and 27.5% (39/142), respectively. Three participants did not complete the PSST questionnaire completely and were excluded. Comparison of demographic characteristics of the study subjects between premenstrual symptom severity groups is presented in Table 1. There was a significant difference between groups in terms of age (*p* = 0.017). The post hoc analysis revealed that the age of the participants in the severe PMS group was significantly lower than participants with mild PMS group (*p* = 0.013) (Table 1).

**Table 1:** Comparison of the demographic characteristics between PSST groups

Variable	Total *N* = 139		Mild *n* = 57		Moderate *n* = 45		Severe *n* = 41		*p*
	Mean	SD	Mean	SD	Mean	SD	Mean	SD	
Age (years)	41.40	7.39	42.85^a^	7.64	41.58	7.95	38.54^a^	5.38	0.017*†

Variable	Total *N* = 143		Mild *n* = 57		Moderate *n* = 45		Severe *n* = 41		*p*
	Frequency	%	Frequency	%	Frequency	%	Frequency	%	
*Education*									
Below high school	17	12.3	8	14.5	6	13.3	3	7.9	0.191‡
High school	24	17.4	8	14.5	11	24.4	5	13.2	
Diploid	6	4.3	4	7.3	1	2.2	1	2.6	
Bachelor	50	36.2	25	45.5	14	31.1	11	28.9	
Masters	27	19.6	6	10.9	10	22.2	11	28.9	
PhD	14	10.1	4	7.3	3	6.7	7	18.4	

^†^One-way analysis of variance (ANOVA) and Tukey post hoc test were used for the comparison

^‡^The Monte Carlo test was used for the comparison

^a^*p* = 0.013

*The difference is significant

There was no significant difference between groups in terms of anthropometric parameters (Table 2). Comparison of nutrient intakes between PMS severity groups is presented in Table 3. There was no significant difference between the groups in terms of nutrient intakes (Table 3).

**Table 2:** Comparison of anthropometric and questionnaire scores between PSST groups

Variable	Total *N* = 139		Mild *n* = 57		Moderate *n* = 45		Severe *n* = 41		*p*
	Mean	SD	Mean	SD	Mean	SD	Mean	SD	
Weight (kg)	70.43	9.12	70.71	10.44	70.04	8.56	70.49	7.80	0.934
Height (cm)	158.62	5.32	157.91	5.50	158.29	4.83	160.03	5.50	0.149
BMI (kg/m^2^)	22.20	2.86	22.49	3.38	22.09	2.66	21.88	2.22	0.588
Waist circumference (cm)	89.60	7.00	90.44	7.95	89.97	6.68	87.95	5.67	0.222
Hip circumference (cm)	105.63	7.41	105.67	9.00	106.21	7.01	104.89	5.03	0.724
Waist-to-hip ratio	0.85	0.05	0.86	0.06	0.85	0.05	0.84	0.04	0.205
Wrist circumference (cm)	15.86	1.29	15.79	1.21	15.96	1.37	15.85	1.33	0.819
Neck (cm)	34.25	2.63	33.97	2.79	34.54	2.45	34.31	2.63	0.556
Mid-arm-circumference (cm)	30.89	3.05	30.81	3.53	31.26	2.83	30.59	2.56	0.596
Quality of life score	91.09	11.18	91.87	9.52	89.71	12.84	91.58	11.44	0.602
QUIKI	0.32	0.02	0.32	0.02	0.33	0.02	0.32	0.03	0.413
Serum vitamin D (ng/mL)	14.97	5.16	14.76	5.53	15.16	4.61	15.05	5.34	0.922†

Variable	Total *N* = 139		Mild *n* = 57		Moderate *n* = 45		Severe *n* = 41		*p*
	Frequency	%	Frequency	%	Frequency	%	Frequency	%	
Vitamin D status									
1st tertile	114	82.0	47	85.5	38	84.4	29	74.4	0.472‡
2nd tertile	23	16.5	7	12.7	6	13.3	10	25.6	
3rd tertile	2	1.4	1	1.8	1	2.2	0	0.0	
Metabolic syndrome	59	41.3	28	50.9	13	28.9	18	46.2	0.110ǂ
CKD stage									
3b	140	99.3	55	100.0	44	97.8	41	100.0	0.639‡
Other	1	0.7	0	0.0	1	2.2	0	0.0	

One-way analysis of variance (ANOVA) and Tukey post hoc test were used for the comparison

^‡^The Monte Carlo test was used for the comparison

ǂThe chi square test was used for the comparison

**Table 3:** Comparison of nutrient intakes between PSST groups

Variable	Total *N* = 139		Mild *n* = 57		Moderate *n* = 45		Severe *n* = 41		*p*
	Mean	SD	Mean	SD	Mean	SD	Mean	SD	
Protein (g)	69.25	33.37	70.08	29.15	70.51	44.90	66.46	21.60	0.863
Fat (g)	82.30	32.24	85.32	34.33	79.51	34.04	81.14	26.88	0.696
SFA (g)	31.06	13.90	32.57	13.09	30.27	15.76	29.78	12.86	0.632
MUFA (g)	30.01	15.53	30.48	17.23	28.08	13.06	31.67	15.84	0.615
PUFA (g)	13.10	6.09	13.75	7.48	12.54	4.57	13.14	5.39	0.533
Trans fatty acid (g)	2.39	1.71	2.61	1.75	2.40	1.87	2.05	1.39	0.368
Cholesterol (mg)	328.03	153.290	320.46	139.33	345.25	179.28	318.42	141.79	0.703
Carbohydrate (g)	221.17	115.23	216.34	73.37	221.62	170.26	231.69	82.15	0.849
Energy (kcal)	1881.99	730.97	1898.42	643.96	1853.11	958.65	1892.46	526.58	0.957
Fiber (g)	20.93	17.75	21.14	9.90	21.75	28.46	19.60	7.51	0.879
Sodium (g)	1543.01	1093.88	1496.54	629.01	1689.69	1720.76	1433.64	534.14	0.587
Potassium (g)	2879.37	1194.38	2961.55	1047.24	2774.98	1403.96	2882.73	1153.85	0.777
Calcium (mg)	738.72	416.16	736.13	353.57	774.49	547.22	697.73	315.51	0.751
Magnesium (mg)	304.01	189.93	308.13	129.18	304.32	283.18	297.37	110.77	0.971
Phosphorus (mg)	1200.61	622.03	1201.75	439.86	1244.22	911.74	1145.43	394.63	0.809
Iron (mg)	10.90	7.16	10.58	4.53	11.69	10.89	10.41	4.18	0.707
Copper (mg)	2.73	1.64	2.52	1.17	2.96	2.11	2.76	1.66	0.461
Zinc (mg)	8.44	4.82	8.37	3.30	8.77	7.24	8.16	2.75	0.866
Chloride (mg)	2626.71	1770.01	2563.77	1027.75	2835.73	2785.03	2465.91	857.61	0.659
Manganese (mg)	5.56	4.46	5.36	2.63	6.05	7.14	5.28	1.55	0.714
Selenium (µg)	63.32	35.17	58.74	25.02	68.93	45.47	63.39	34.06	0.418
Iodine (µg)	97.51	63.74	102.75	75.33	92.70	52.36	95.47	58.43	0.757
Retinol (µg)	434.69	230.55	407.37	203.02	472.44	270.58	429.83	217.405	0.433
Carotene (µg)	1219.17	908.98	1192.99	754.31	1129.35	863.27	1368.98	1158.12	0.539
Vitamin D (µg)	1.39	0.71	1.42	0.78	1.40	0.76	1.35	0.57	0.920
Vitamin E (mg)	11.47	7.35	11.19	8.88	10.97	6.11	12.49	6.21	0.662
Thiamin (mg)	1.46	0.78	1.48	0.57	1.45	1.11	1.43	0.55	0.964
Riboflavin (mg)	2.34	1.21	2.22	0.94	2.54	1.39	2.27	1.34	0.461
Niacin (mg)	16.85	11.30	16.55	7.74	17.75	16.99	16.19	6.37	0.830
Vitamin B6 (mg)	6.69	5.04	5.92	3.24	7.65	6.31	6.69	5.49	0.293
Vitamin B12 (µg)	17.47	13.36	15.41	8.86	20.26	16.51	17.20	14.53	0.250
Folate (µg)	274.72	134.01	273.28	97.89	281.92	189.43	268.09	98.91	0.910
Panthotenate (mg)	6.98	3.23	6.83	2.59	7.21	4.12	6.92	3.23	0.861
Biotin (µg)	58.81	29.40	57.62	25.71	59.95	35.71	59.28	26.86	0.933
Vitamin C (mg)	214.59	146.54	232.35	171.13	185.13	97.27	223.77	155.10	0.312

One-way analysis of variance (ANOVA) and Tukey post hoc test were used for the comparison

Linear regression analysis was performed to assess the relationship between study variables and PSST score. The adjusted *R* square for the selected model was 0.274 and the *R* square was 0.361 for the model. The relationship between selected study variables and PSST questionnaire score is shown in Table 4. There was a significant relationship between PSST score and age (*p* = 0.001), depression (*p* = 0.007), vitamin D status (*p* = 0.011), and polyunsaturated fatty acid (PUFA) (*p* = 0.040).

**Table 4:** Relationship between PSST score and selected study variables

Variable	Unstandardized coefficients		Standardized coefficients	*t*	Sig.
	*B*	Std. error	Beta		
Age	− 0.537	0.158	− 0.317	− 3.398	0.001*
Depression	0.484	0.176	0.350	2.746	0.007*
Anxiety	− 0.040	0.247	− 0.020	− 0.162	0.872
Stress	0.011	0.055	0.019	.197	0.844
Vitamin D category	6.229	2.391	0.226	2.605	0.011*
PUFA (energy adjusted)	− 10.516	5.042	− 0.311	− 2.086	0.040*
MUFA (energy adjusted)	7.702	4.880	0.234	1.578	0.118
Vitamin E (energy adjusted)	3.754	3.417	0.167	1.099	0.275
PAB	0.033	0.022	0.135	1.502	0.136
SFA (energy adjusted)	− 5.966	5.830	− 0.173	− 1.023	0.309
Trans fatty acid (energy adjusted)	− .426	2.496	− 0.022	− .171	0.865
Cholesterol (energy adjusted)	3.765	2.564	0.142	1.468	0.145
WHR	− 19.392	22.204	− 0.081	− .873	0.385

Linear logistic regression was used for the analysis

*Significant relationship

Multinomial logistic regression was used to assess the relationship between selected study parameters and premenstrual syndrome severity categories (Table 5). The Nagelkerke’s pseudo-*R* square for the model was 0.402. There was a significant relationship between moderate PMS severity and energy-adjusted saturated fatty acid (SFA) (*p* = 0.018, OR = 0.010 and 95% CI for OR: < 0.001 and 0.452), and energy-adjusted riboflavin (*p* = 0.042, OR = 0.005, 95% CI for OR: < 0.001 and 0.821) and between severe PMS severity and age (*p* = 0.034, OR = 0.906, 95% CI for OR: 0.826 and 0.993), and energy-adjusted monounsaturated fatty acid (MUFA) intake (*p* = 0.041, OR = 23.789, 95% CI for OR: 1.138 and 497.294).

**Table 5:** Relationship between selected study parameters and premenstrual syndrome severity categories

Severity	Variable	*p*	OR	95% CI for OR	
				Lower	Upper
Moderate	Age	0.959	0.998	0.925	1.076
	Fiber (energy adjusted)	0.610	1.770	0.198	15.855
	SFA (energy adjusted)	0.018*	0.010	< 0.001	0.452
	MUFA (energy adjusted)	0.087	11.417	0.704	185.053
	PUFA (energy adjusted)	0.553	0.431	0.027	6.928
	Trans fatty acid (energy adjusted)	0.255	2.322	0.545	9.893
	Vitamin E (energy adjusted)	0.553	1.762	0.272	11.431
	Riboflavin (energy adjusted)	0.042*	0.005	< 0.001	0.821
	Niacin (energy adjusted)	0.218	11.249	0.238	530.796
	Vitamin B6 (energy adjusted)	0.855	1.479	0.022	97.481
	Vitamin B12 (energy adjusted)	0.455	0.208	0.003	12.833
	Panthotenate (energy adjusted)	0.621	3.321	0.028	388.803
	Vitamin C (energy adjusted)	0.335	1.538	0.641	3.691
	Depression	0.515	1.033	0.936	1.140
	Anxiety	0.892	0.991	0.865	1.134
	Stress	0.118	1.066	0.984	1.154
Severe	Age	0.034*	0.906	0.826	0.993
	Fiber (energy adjusted)	0.696	1.603	0.151	17.066
	SFA (energy adjusted)	0.172	0.064	0.001	3.320
	MUFA (energy adjusted)	0.041*	23.789	1.138	497.294
	PUFA (energy adjusted)	0.096	0.082	0.004	1.558
	Trans fatty acid (energy adjusted)	0.617	0.683	0.153	3.052
	Vitamin E (energy adjusted)	0.168	4.098	0.553	30.401
	Riboflavin (energy adjusted)	0.446	0.094	0.000	41.236
	Niacin (energy adjusted)	0.491	4.286	0.068	269.970
	Vitamin B6 (energy adjusted)	0.754	2.153	0.018	261.690
	Vitamin B12 (energy adjusted)	0.740	0.497	0.008	30.948
	Panthotenate (energy adjusted)	0.997	0.992	0.009	105.529
	Vitamin C (energy adjusted)	0.936	1.037	0.432	2.487
	Depression	0.409	1.044	0.943	1.155
	Anxiety	0.455	0.947	0.820	1.093
	Stress	0.061	1.081	0.996	1.173

*The difference is significant

## Discussion

We evaluated the relationship between PSST score and biochemistry and anthropometric indices. We used the PSST questionnaire, which rates the impact of premenstrual symptoms on daily activities. Another usage of PSST is to screen for premenstrual dysphoric disorder. The mean PSST Score in our study was 20.46 $\pm$12.05. Study participants were classified into three groups based on PSST cut-offs. Majority of the participants (38.7%) had mild PMS symptoms, while moderate and severe PMS symptoms were observed in 31.7% and 27.5% of the participants, respectively. We also compared the demographic characteristics of participants in the different PMS categories and found that individuals with severe PMS were younger than individuals with those with mild PMS (*p* = 0.017).

We found no significant difference in anthropometric parameters between PMS severity groups. The definition for abdominal obesity was WC > 80 cm. Based on a recent systematic review and meta-analysis in Iran the cut off for abdominal obesity was 89.24 cm in women [39]. Therefore, based on either reference range the participants in our study had abdominal obesity. This finding was predictable as all participants had abdominal obesity based on national cut off. Therefore, it can be inferred that subjects with severe PMS symptoms had lower WC and BMI. Although this difference was not statistically significant, having a mean WC below 89.24 cm indicates a clinical significance. Therefore, the findings of our study indicated that participants with severe PMS had a lower degree of abdominal obesity although there was no clinical or statistical difference between groups in terms of BMI.

It was previously shown that BMI and PMS severity were linearly correlated [40, 41]. A study showed that women with BMI higher than 27.5 kg/m^2^ had a higher chance of developing severe PMS after 10 years compared to women with BMI below 20 kg/m^2^ [40]. This finding was in contrast to the findings of our study. However, our study did not include obese or underweight women based on BMI categories. While the BMI of the participants in our study was in the normal range, there was an inverse relationship between PMS severity and WC and BMI. This relationship might be attributed to lack of subjects with high BMI and WC. Similarly, a study found a U-shape relationship between BMI and PMS [42]. A possible reason for the relationship between BMI and PMS symptom severity might be the lower level of estradiol in women with adiposity compared to normal-weight women [40, 43]. This hypothesis may not be tested in our study because sex hormones were not assessed for study subjects.

Our study indicated a significant relationship between age and PSST score indicating that younger individuals were more likely to experience more severe PMS symptoms. It was previously shown that younger women experience more severe PMS symptoms [44–46]. These findings were in line with our study findings. Hypothetically PMS should increase with age; therefore, external factors were presented as the causes of high experience of severe PMS among younger women [47]. The relationship between younger age and more severe PMS might be due to the high prevalence of mental disorders, including depression, among younger women [47]. Our findings indicated a significant relationship between depression and PSST score, which might be an indicative of the role of depression in severity of PMS among younger women. Previous studies also indicated that depression during the luteal phase might aggravate PMS severity [48–52]. As our study was cross-sectional, we could not indicate causation. Therefore, our study findings could not document whether PMS causes depression or depression aggravates PMS.

We found a significant relationship between serum vitamin D tertiles and PSST score indicating lower Vitamin D intakes in women with more severe PMS symptoms. This finding was in line with the findings of a previous study [53]. This effect might be related to the anti-inflammatory effects of vitamin D [54].

No significant difference in crude nutrient intakes was found between PMS severity groups. This finding could be due to effect of other confounders especially energy intake. Therefore, the regression analysis was performed using the energy-adjusted nutrient intakes.

Based on the findings of multinomial logistic regression, higher SFA intake was less likely to develop moderate PMS symptoms, while higher MUFA intake was related to severe PMS symptoms, which were in line with the findings of a previous study [45]. We found a significant relationship between PMS and PUFA. In contrast to the findings of our study, a previous study indicated no significant relationship between PUFA intake and PMS [45]. Currently, no documented mechanism has been suggested for the relationship between PMS severity and SFA, MUFA, and PUFA intake [45]. As the regression analysis adjusts the variables, including BMI and central adiposity, the results of our study suggested that type of fatty acid intake, but not total fat intake, can be an independent risk factor for PMS severity.

We reported a significant association between PMS and riboflavin, which was similar to the findings of a previous study [46]. The possible mechanism for the effect of riboflavin on PMS severity is its role in inflammation and pain pathway [55, 56].

One of the limitations of the current study was not evaluating serum estradiol and the presence of polycystic ovarian syndrome, which were not possible due to financial limitations. We included 139 cases in our cross-sectional study. In several studies with the same method as us, the sample size was much bigger. Therefore, we mention that our study sample size could be bigger, and we could have a more reliable result. The cross-sectional study gives us an overview of our subject. But outcomes of cross-sectional studies should be evaluated by more accurate studies like case–control, cohorts, or RCT. We could not find a significant association between some variables and PMS, but some studies reported an association between this variable. Hence, the prevalence of some confounding factors in our study disrupted our findings.

For future research, we suggest a larger sample size for studies, at least 800. Therefore, a more accurate result can be obtained. Researchers can commit case–control or cohort studies for more accurate results. Some confounding factors spoil the result, and we recommend deleting these factors.

## Conclusion

We have investigated the factors associated with PMS severity. A younger age was associated with a greater PMS severity. Some nutrition factors were associated with PMS severity. A higher intake of some nutrients like riboflavin, SFA, and vitamin D were associated with a lower risk of having a high severe PMS. Factors like PUFA and MUFA may be related to PMS severity.

## Data Availability

The data that support the findings of this study are available from the corresponding author upon reasonable request.
